# Behavior, Ecology and Integrated Management of Fruit Flies

**DOI:** 10.3390/insects17050521

**Published:** 2026-05-20

**Authors:** Marc De Meyer, Nikos T. Papadopoulos

**Affiliations:** 1 Department of Zoology, Royal Museum for Central Africa, Leuvensesteenweg 13, B3080 Tervuren, Belgium; 2 Laboratory of Entomology and Agricultural Zoology, Department of Agriculture, Crop Production and Rural Environment, University of Thessaly, Fytokou St, GR38446 Volos, Greece

## 1. Fruit Flies—Importance and Invasion Dynamics

Invasive species, whose geographic distribution is expanding, seeing introduction and establishment in previously pest-free areas, have major environmental and economic impacts. The problem of invasive pests is multidimensional and complex and can only be tackled through strong integration and the use of various approaches [1]. Climate change, intense human mobility, and increased international and transcontinental trading have brought biological invasions to the forefront of the list of threats to agricultural production worldwide.

Fresh-fruit-and-vegetable production and trading are among the many sectors suffering considerable losses worldwide because of these invasive pests. True fruit flies, species of the large Dipteran family Tephritidae (comprising more than 5000 species [2]), constitute perhaps the most important group of pests affecting fresh fruits and vegetables, including numerous major invasive pests that affect food production and incur high economic losses annually [3]. The Mediterranean fruit fly (medfly), the Oriental fruit fly (OFF), and the peach fruit fly (PFF) are three of the most important invasive fruit fly species [4]. The medfly was introduced in the Mediterranean basin in the 19th century and has been established in the region ever since. It is the target of major eradication campaigns in many areas (e.g., North America, Australia, and Chile), and it is frequently detected in California, where a “preventive” eradication project based on releases of sterile males has been ongoing for a couple of decades [5]. Because of climate change, during the past few decades, more-temperate areas of the Northern Hemisphere are gradually warming and becoming suitable for this species, resulting in a spread northward [6]. The other two pests (the OFF and PFF) have expanded their geographic distributions to areas neighboring Europe and frequently arrive in Europe via infested fruits [7]. Accordingly, the EU has included them among the 20 priority pests (being two of the four fruit fly species on this list) in the Delegated EU Regulation 2019/1702. Each EU member state is required to draw up an action plan in order to avoid introduction into the region. Nevertheless, in recent years, there have been reports of both species in European countries, and in some areas, the number of sightings is considered high, with annual recurrence [8]. Several European countries have reported detections and are experiencing invasive-fruit-fly outbreaks (e.g., Austria, Belgium, France, Greece, Italy, Slovakia, and Spain—EPPO global database, https://gd.eppo.int/taxon/DACUDO/reporting accessed on 8 May 2026).

## 2. The Horizon 2020-Funded FF-IPM Project

As a further result, the EU’s Horizon 2020 programme launched a call in 2017 addressing the topics of food security, sustainable agriculture, and forestry; marine, maritime, and inland water research and the bioeconomy; and, more specifically, new and emerging risks to plant health in Europe. The University of Thessaly (Volos, Greece), under the coordination of Prof. Nikos Papadopoulos, successfully obtained a grant to address the issue, focusing on fruit flies in particular. Together with 21 other partners from 17 countries and 71 scientists within the consortium, it initiated the project entitled “FF-IPM—In silico boosted, pest prevention and off-season focused IPM against new and emerging fruit flies” on 1 September 2019 to tackle a wide range of objectives over the next 54 months. The aim of the project was to introduce “in silico”-supported prevention, detection, and Integrated Pest Management (IPM) approaches for both new and emerging fruit flies based on spatial modelling across a wide range of spatial levels, novel decision support systems, and new knowledge regarding the biological traits of the target species, fruit trading, and socioeconomics. It addressed the following specific objectives:

✓ Understand the factors that determine the success of biological invasions in the context of climate change.
✓ Prevent insect invasion though the use of innovative tools that prevent the introduction of infested fruits and locate populations in the early stages of an invasion.
✓ Manage established species in out-of-season periods through biological control.
✓ Develop new strategies based on the use of thorough ecological modelling and appropriate hardware and software.
✓ Achieve in silico boosting of current IPM tools.
✓ Help maintain the productivity and sustainability of the European fruit production industry.

By its conclusion in February 2024, the FF-IPM project had generated numerous important outputs, providing new insights into fruit fly invasion and offering novel tools and approaches to addressing fruit fly invasions in Europe (see the Layman’s report of the project at https://fruitflies-ipm.eu/news-and-events/laymans-report/ accessed on 8 May 2026). Several novel detection and interception tools—such as electronic traps, mobile applications that aid in identification, early detection systems, electronic ‘e-noses’ that can distinguish infested fruits from non-infested ones based on volatiles, and molecular ID tools—had been developed or improved. The project introduced the novel “off-season” approach in fruit fly management that has ushered in a paradigm shift in the control of other pests. It also resulted in the advancement, development, and testing of Integrated Pest Management tools that can be used both “off-” and on-Season, such as such as entomopathogenic fungi or nematodes and predator-based biocontrol approaches. To execute the “off-” and “on-Season” management strategy, a virtual-farm decision support and service (DSS) system was developed. The FF-IPM project developed bioclimatic and population growth models for the target species (*Ceratitis capitata*, *Bactrocera dorsalis*, and *Bactrocera zonata*) that can support interception (e.g., estimating the risk of infestation for imported commodities), detection, eradication, and containment efforts. Communication and dissemination of the new information gathered, geared towards stakeholders and end-users (particularly farmers and NPPOs), was considered a major topic. To this end, several workshops and webinars were organized, and a wide variety of modern communication channels were exploited in order to spread the information as far as possible. In addition, there was also substantial and important scientific output, including several articles published in peer-reviewed journals.

## 3. Highlights of the Special Issue

The suggestion made by the editorial office of the MDPI journal *Insects* to initiate a Special Issue on “Fruit Flies (Diptera: Tephritidae): Behavior, Ecology and Integrated Management” was considered an appropriate way to offer our colleagues a chance to make some of their findings publicly available to a wider audience in a coordinated fashion. In addition, it was also a chance to highlight some of the complementary activities conducted by peers in the field through other research programs in other parts of the world. The result is a Special Issue containing 10 articles covering all aspects related to state-of-the-art fruit fly research.’

Articles such as Bjeliš et al.’s (on the invasion history and dispersion dynamics of *Ceratitis capitata* in the Balkans) or Souza Coelho et al.’s (on trophic interactions of fruit flies in Minas Gerais, Brazil) give an insight into the ecological aspects that affect fruit fly species’ invasion potential, while Kokkari et al.’s work (on the behavioral effect of fruit volatiles on *Bactrocera oleae*) focuses on the ethological drivers of fruit infestation. The development and application of novel detection techniques are highlighted in the papers by Balan et al. (on applying real-time PCR to *Zeugodacus cucurbitae* and closely related species), Vanbergen et al. (on the genomic tracing of *Bactrocera dorsalis* detections in Belgium), and Anastasaki et al. (on using electronic nose systems to intercept fruit-fly-infested fruit), while Wimbush et al.’s work (on using quantum dots as a marking technique) explores a novel technique involving the use of field research to track movement and dispersion patterns. More practical aspects can be found in the papers by Opoku et al. (on the regulatory pest management of fruit flies in Ghana), Siden-Kiamos et al. (on control by disruption of bacterial symbionts), and Junaid Nisar et al. (on the improvement of attractants via phagostimulants).

## 4. Conclusions

In all, we hope that this Special Issue provides a ‘taste’ of the diversity of fruit-fly-related research, which is ongoing and will undoubtedly be relevant in the years to come. Fruit flies will remain at the top of list of pests of horticultural commodities, and it seems invasion events will continue challenging the production and trade of fresh fruits and vegetables. The ongoing fruit fly invasion in Europe is an example of how science and major research initiatives such as the FF-IPM project foresee emerging challenges and, through a structured approach, can greatly contribute to preparedness and management.
